# Microbiome-Associated Drug Response Variability in Heart Failure Treatment

**DOI:** 10.3390/life16050823

**Published:** 2026-05-15

**Authors:** Andrea Rab, Annamária Magdás, Attila Frigy

**Affiliations:** 1 Doctoral School of Medicine and Pharmacy, George Emil Palade University of Medicine, Pharmacy, Science and Technology of Targu Mures, 540142 Targu Mures, Romania; 2 Department of Internal Medicine IV, George Emil Palade University of Medicine, Pharmacy, Science and Technology of Targu Mure, 38 Gheorghe Marinescu Street, 540142 Targu Mures, Romania; 3 Department of Internal Medicine, Clinical County Hospital Mures, 1 Gheorghe Marinescu Street, 540103 Targu Mures, Romania; 4 Department of Cardiology, Clinical County Hospital Mures, 1 Gheorghe Marinescu Street, 540103 Targu Mures, Romania

**Keywords:** gut microbiome, heart failure, metabolites, cardiovascular medication, pharmacomicrobiomics, personalized medicine

## Abstract

Gut microbiome composition influences cardiovascular drug efficacy and safety, yet its integration into heart failure (HF) management remains underexplored. Alterations in intestinal microbial communities have been linked to atherosclerosis, coronary artery disease, heart failure, and hypertension through multiple mechanisms. Dysbiosis disrupts the balance between commensal and pathogenic bacterial species, impairing gut barrier function and activating inflammatory pathways. The altered microbial ecosystem modulates the production of key metabolites—such as trimethylamine-N-oxide (TMAO), short-chain fatty acids (SCFAs), and secondary bile acids (BAs)—that directly impact cardiovascular function. This narrative review synthesizes current evidence on bidirectional interaction between heart failure pharmacotherapy and gut microbiome composition. Commonly used drugs in heart failure management show microbiome-dependent pharmacokinetics. Digoxin undergoes bacterial inactivation by *Eggerthella lenta*, while angiotensin converting enzyme inhibitors and beta-blockers demonstrate enhanced efficacy with specific *Firmicutes* populations. Conversely, certain probiotic strains attenuate drug-induced gut barrier injury and restore gut homeostasis. Sodium–glucose cotransporter 2 inhibitors (SGLT2i), mineralocorticoid receptor antagonists, and angiotensin receptor–neprilysin inhibitors exhibit beneficial microbiome-modulating effects beyond their primary cardiovascular actions. These findings underscore the potential for microbiome-informed precision medicine in heart failure. However, significant methodological challenges must be addressed, including lack of standardization in microbiome profiling, small sample sizes, and limited longitudinal data. Future research should focus on identifying specific microbial signatures that predict drug response, developing targeted probiotic interventions, and conducting prospective clinical trials to validate pharmacomicrobiomics approaches in heart failure management.

## 1. Introduction

**Figure 1:**
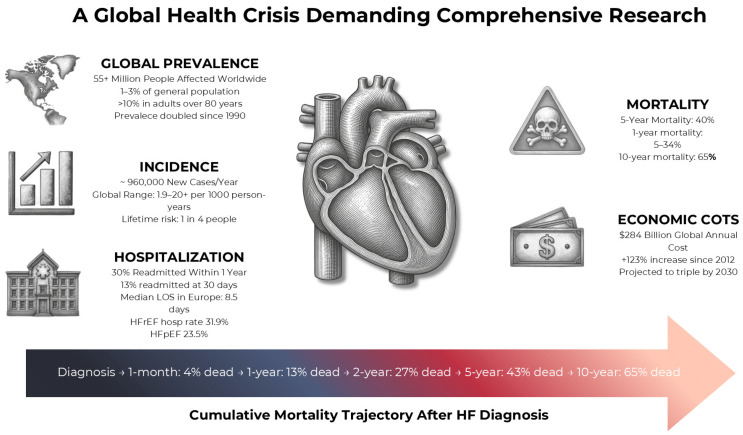
The Global Burden of Chronic Heart Failure. This infographic synthetizes key epidemiological indicators of the heart failure burden, illustrating the magnitude of disease prevalence, incidence, healthcare utilization, mortality and economic burden. The figure comprises five main panels and a cumulative mortality timeline. Abbreviations: HF heart failure; HFrEF heart failure with reduced ejection fraction; HFpEF heart failure with preserved ejection fraction; LOS length of stay [1].

Despite significant advances in cardiology, heart diseases remain the leading cause of death worldwide [1,2] (Figure 1). While established risk factors contribute to the development of cardiovascular disease (CVD), new research suggests that changes in the gut microbiome community may play an additional role in the occurrence and progression of cardiovascular pathologies, including heart failure (HF) [3,4].

Decreased cardiac output and systemic congestion in heart failure are associated with compromised intestinal function due to epithelial wall edema and impaired bowel perfusion leading to barrier integrity weakening and microbial translocation into the circulation. These mechanisms in turn cause endotoxemia and enhanced systemic inflammation [5].

Furthermore, patients with heart failure exhibit a different microbiota composition compared to healthy individuals. Observational studies report a significantly reduced alpha-diversity, and a distinct metabolomic and lipid profile compared to healthy controls [6,7]. The overgrowth of pathogenic bacteria, such as *Escherichia*/*Shigella*, *Campylobacter* and *Salmonella*, has been reported in chronic heart failure and subsequently related to the severity of intestinal permeability [8].

Recent advances point out that bacterial enzyme activity may influence drug metabolism and efficacy, while drugs have the potential to change microbial landscape and metabolite function. Understanding the interdependent relationship between cardiovascular pharmaceuticals and microbiome activity is essential for comprehensive management of heart failure [9]. Emerging studies indicate that several drugs, such as antihypertensives, cholesterol-lowering substances, and antithrombotic medications, interact with gut microbial composition and diversity [10]. Observational findings suggest that statins may have implications in reducing systemic inflammation in obese patients by promoting the growth of various beneficial bacterial phyla [11]. Likewise, agents targeting the renin–angiotensin–aldosterone system (RAAS), beta-blockers and diuretics may also alter microbiome composition [12]. On the other hand, changes in the gut microbial profile may alter drug metabolism, affecting the efficacy and safety of antithrombotic therapies [13].

While associations between heart failure medication and microbiome changes are increasingly recognized, the underlying mechanisms remain unclear, requiring further research. It is proposed that the bidirectional interaction between cardiovascular drugs and gut microbial profiles has a significant role in modulating treatment response. Thus, the aim of this review is to integrate dispersed mechanistic interplay between cardiovascular pharmacotherapy and gut microbiome activity and microbial metabolites modulation, to foreground underexplored areas due to limited causal human data, scarce longitudinal and dose–response studies, lacks on polypharmacy and microbiome–pharamacogenomic interactions, as well as the heterogeneity of the existing methods and analyze potential microbiome-guided strategies in heart failure management.

## 2. Methods

### 2.1. Search Strategy

We conducted a comprehensive literature search across three major databases—PubMed/MEDLINE, Web of Science, and Scopus—from January 2014 to March 2026. This timeframe captures the modern era of microbiome research following advances in next-generation sequencing technologies.

The following search terms were included: (“gut microbiome” OR “gut microbiota” OR “intestinal microbiome” OR “intestinal flora”) AND (“heart failure” OR “cardiac failure” OR “HF” OR “HFpEF” OR “HFrEF”) AND (“drug” OR “medication” OR “pharmacotherapy” OR “treatment” OR “therapy”) AND (“metabolism” OR “bioavailability” OR “efficacy” OR “response” OR “interaction”).

Additional searches targeted specific drug classes and microbiome-derived metabolites.

### 2.2. Inclusion and Exclusion Criteria

Inclusion criteria:

- Clinical studies (observational, interventional) and preclinical studies (animal model, in vitro).
- Studies examining gut microbiome composition in heart failure patients.
- Studies investigating interactions between cardiovascular medications and gut microbiota.
- Studies evaluating microbiome-derived metabolites in heart failure.
- Articles published in English in peer-reviewed journals.

Exclusion criteria:

- Case report with fewer than 5 patients.
- Conference abstracts without full-text availability.
- Articles lacking microbiome analysis or cardiovascular drug evaluation.

Following the selection process, a total of 105 articles were included in this narrative review. The selected studies encompassed clinical and preclinical investigations examining gut microbiome composition in heart failure, interactions between cardiovascular medications and gut microbiota, and the role of microbiome-derived metabolites in heart failure pathophysiology and pharmacotherapy.

The 105 included articles comprised the following study types: 76 original research articles (human observational and cross-sectional studies), 9 primary animal/preclinical studies (in vivo animal models and in vitro experiments), 4 mixed studies (combining both animal and human components), 6 review articles, 4 clinical interventional studies, 3 book chapters, 2 systematic reviews or meta-analyses, and 1 clinical guideline. The inclusion of both human and preclinical studies reflects the current evidence base, in which mechanistic insights from animal models complement and contextualize clinical findings in HF pharmacomicrobiomics.

## 3. The Gut–Heart Axis—Microbiome in Heart Failure

The human gut hosts an average of 1000 to 1150 microbial strains [14]. Trillions of commensal microorganisms residing in the intestinal lumen—in varying proportions—constitute a complex organ that plays a crucial role in maintaining gut barrier integrity, metabolic homeostasis, and the inflammatory response [15]. Large-scale populational studies, including the European Metagenomics of the Human Intestinal Tract (MetaHIT) and the US-led Human Microbiome Project, managed to provide a characterization of different microbial compositions in health and disease [16,17].

Although challenges have arisen in identifying a “healthy” gut microbiome composition due to the high inter-individual variability, it has been shown that in healthy states the gut microbial ecosystem remains relatively stable. Approximately 90% is dominated by the *Firmicutes* and *Bacteroides* species, while *Proteobacteria*, *Actinobacteria*, *Cyanobacteria*, *Fusobacteria*, and *Verrucomicrobia* comprise the majority of the remaining fraction [18]. These species are responsible for maintaining the intestinal mucosal barrier, enhancing digestion, supplying nutrients, preventing infections, and supporting normal coagulation [19].

Heart failure induces profound alterations in gut microbiome composition and diversity. Multiple studies have demonstrated that HF patients exhibit significantly reduced alpha-diversity compared to healthy controls [6,15,16]. At the phylum level, HF patients show a characteristic dysbiotic pattern. The *Firmicutes*-to-*Bacteroidetes* ratio is frequently altered, though the direction of change varies across studies [16]. More consistently, HF is associated with increased abundance of potentially pathogenic taxa, including *Proteobacteria* (particularly *Enterobacteriaceae* family), *Streptococcus*, and *Veillonella* species [8,18]. Conversely, beneficial commensal bacteria such as *Faecalibacterium prausnitzii*, *Roseburia*, and *Eubacterium* species are often depleted in HF patients [19].

The mechanisms underlying microbiome alterations in HF are multifactorial. Reduced cardiac output leads to intestinal hypoperfusion and congestion, causing bowel wall edema and epithelial barrier dysfunction [5,20]. The resulting “leaky gut” facilitates endotoxin translocation, metabolite and cytokine influx into systemic circulation. This can trigger dysbiosis-related systemic inflammation, which has been implicated in cardiovascular pathogenesis [21,22]. A self-promoting cycle can emerge in which inflammatory pathways—including the expansion of cardiac myosin-specific Th17 cells, toll-like receptor signaling, and increased production of proinflammatory cytokines such as tumor necrosis factor-α (TNF-α) and interferon-γ (IFN-γ)—further compromise gut barrier function [23].

## 4. Role of the Key Gut-Derived Metabolites

**Figure 2:**
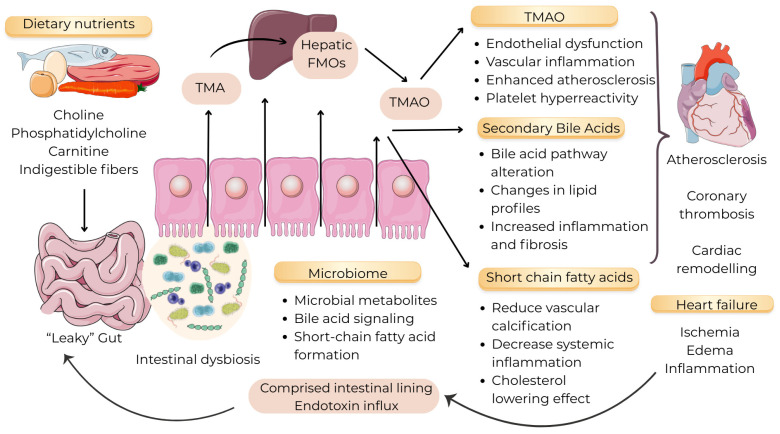
Gut microbiome–heart axis. Dietary precursors (choline, phosphatidylcholine, carnitine, indigestible fibers) are metabolized by the intestinal microbiota to produce microbial metabolites such as trimethylamine (TMA) and short-chain fatty acids (SCFAs). TMA is absorbed and oxidized in the liver by flavin-containing monooxygenases (FMOs) to form trimethylamine N-oxide (TMAO). Elevated TMAO promotes endothelial dysfunction, vascular inflammation, enhanced atherosclerosis, and platelet hyperreactivity. Microbiome-mediated bile acid transformations generate secondary bile acids that alter bile acid signaling, modify lipid profiles, and increase inflammation and fibrosis. Conversely, SCFAs provide protective effects by reducing vascular calcification, decreasing systemic inflammation, and exerting cholesterol-lowering actions. Intestinal dysbiosis and increased intestinal permeability (“leaky” gut) facilitate endotoxin influx and systemic inflammatory pathways, creating a feed-forward loop that exacerbates cardiovascular pathology (atherosclerosis, coronary thrombosis, cardiac remodeling) and contributes to the development of heart failure (ischemia, edema, inflammation). Arrows indicate direction of metabolite flux and signaling between diet, gut microbiota, liver metabolism, and vascular and cardiac tissues. (Image provided by Servier Medical Art (https://smart.servier.com/) (accessed on 12 April 2026), licensed under CC BY 4.0 (https://creativecommons.org/licenses/by/4.0/) (accessed on 12 April 2026).

Different microbiota species produce a variety of bioactive compounds, depending on the host’s individual microbiome composition and dietary habits. Emerging evidence suggests that key metabolites implicated in cardiovascular pathogenesis include trimethylamine-N-oxide (TMAO), short-chain fatty acids (SCFAs), and secondary bile acids (SBAs). These metabolites modulate vascular inflammation, lipid and glucose homeostasis, blood pressure regulation and thrombosis risk, underlining the significance of the gut–heart axis [24]. (Figure 2).

### 4.1. Trimethylamine-N-Oxide

TMAO is produced in the liver by flavin monooxygenases from TMA, which gut microbial TMA lyases derive from dietary phosphatidylcholine, choline, and carnitine, found in eggs, fish and red meat. TMA-producing bacteria detected in the human gut were identified as *Escherichia fergusonii*, *Clostridium sporogenes*, and *Proteus penneri* [25].

TMAO has emerged as one of the most extensively studied gut microbiome-derived metabolites in cardiovascular disease [26]. A recent meta-analysis revealed a 67% risk of CVD development linked to high TMAO levels [27]. Accordingly, the association between increased TMAO levels and the risk of major adverse cardiac events (MACE) has been suggested by numerous animal and human experiments [28,29,30]. By altering cholesterol metabolism—either promoting lipid deposition in the arterial walls or inhibiting reverse cholesterol transport—TMAO may contribute to the development of atherosclerosis [31]. Furthermore, high levels of TMAO are linked to the worsening of vascular inflammation, endothelial dysfunction and foam cell formation, subsequently facilitating atherosclerotic plaque development and progression [32,33].

Findings from observational studies indicate that elevated plasma TMAO levels are associated with an advanced cardiometabolic risk profile, including low phospholipid levels and hypomethylation patterns, lower HDL cholesterol and increased incidences of diabetes. Higher TMAO concentrations correlate positively with increased intima-media thickness as an early marker of atherosclerosis, regardless of age, sex or visceral fat mass [34,35].

The potential role of TMAO in development of atherosclerosis and adverse cardiovascular outcomes was further suggested by a multicenter study including 134 participants at high risk for cardiovascular events, that found a strong correlation between elevated TMAO serum levels and subclinical myocardial damage indicated by increased high-sensitivity cardiac troponin-T levels, even after adjusting for traditional cardiovascular risk factors [36].

While these studies established a significant link between TMAO and atherosclerosis independent of other risk factors, the lack of detailed dietary and microbiome data, along with the moderate nature of the intervention, presents limitations in fully understanding the mechanisms and broader applicability of the findings. Several limitations are acknowledged, including potential selection bias due to data availability, the inability to fully rule out recent dietary intake affecting TMAO levels, the cross-sectional nature of the measurements precluding prognostic assessment over time, and a relatively small sample size for patients with multiple risk factors.

On the other hand, longitudinal studies have shown that elevated plasma TMAO levels have prognostic value for arterial thrombosis, stroke and myocardial infarction [37,38]. Landmark research, involving two independent prospective cohorts—Cleveland (530 patients) and Swiss (1683 patients) cohorts—revealed that elevated TMAO levels at presentation were independently associated with a significantly increased risk of MACE and long-term mortality. In the Cleveland cohort, TMAO levels in the highest quartile were correlated with a 30-day MACE risk and a 7-year mortality risk, even in troponin-negative patients. In the Swiss cohort, TMAO also predicted enhanced MACE risk. These findings establish TMAO as a robust, independent prognostic marker for adverse cardiovascular outcomes in acute coronary syndrome patients, suggesting potential clinical utility in risk stratification and highlighting TMAO’s modifiable nature as a target for therapeutic interventions [39].

In STEMI patients, significant increases in TMAO levels were noted from the acute phase to the chronic phase after secondary prevention therapies. Chronic-phase TMAO levels emerged as strong independent predictors of future cardiovascular events and coronary plaque progression. However, limitations of this research occurred due to a smaller-than-required sample size, the absence of a control group, and missing data on left ventricular function [40]. For patients suspected of functional coronary artery disease, elevated levels of TMAO and its precursors were associated with an increased risk of all-cause and cardiovascular death, yet the diagnostic accuracy for the disease was limited. This study also faced challenges such as potential patient misclassification and a lack of dietary information, which may affect the generalizability of its results [41].

Patients with chronic heart failure following a myocardial infarction revealed elevated TMAO levels as an independent predictor of MACE and all-cause mortality, as found in a multicenter prospective cohort study evaluating 1208 chronic heart failure patients with a history of MI. These findings suggest incorporating TMAO into traditional risk factor models in order to enhance risk prediction ability [42].

Furthermore, in a pooled analysis of 11,768 participants in two large, prospective, community-based cohorts—the Cardiovascular Health Study (CHS) and the Multi-Ethnic Study of Atherosclerosis (MESA)—researchers found that higher concentrations of TMAO are independently associated with an increased, 15% risk of incident heart failure. However, when adjusting for renal function, the significance of TMAO diminished, indicating that renal function could mediate these relationships. The study also found similar associations across HF subtypes [43].

In addition, heart failure patients exhibit higher TMAO plasma concentration than healthy individuals, and they positively correlate with B-type natriuretic peptide (BNP) levels, placing TMAO in the role of a potential biomarker for predicting heart failure risk [44].

### 4.2. Short-Chain Fatty Acids

SCFAs—including acetate, propionate, and butyrate—synthesized during the fermentation of indigestible dietary fibers—are among the most well-defined, beneficial microbiome-derived metabolites [2,45]. The main gut bacteria responsible for converting complex carbohydrates into monosaccharides include *Bacteroides*, *Bifidobacterium* and *Faecalibacterium* spp. SCFAs have been established to contribute significantly in sustaining eubiosis, enhancing lipid and glucose metabolism, modulating immune responses, preserving intestinal mucosal integrity, and preventing bacterial translocation into systemic circulation [33,34]. Butyric acid exhibits anti-inflammatory properties and increases nitric oxide concentrations, thereby reducing atherosclerotic burden. Propionic acid supports metabolic homeostasis, while acetic acid helps regulate blood pressure and modulates lipid profiles [46,47].

High propionate levels were negatively correlated with vascular calcification, as was shown in a study that combined a clinical observational cohort, including 92 patients, with in vivo animal experiments, using rat models. Propionate administration was found to ameliorate vascular calcification in rats, improve intestinal barrier function by increased crypt depth and goblet cell count, alongside decreased inflammatory markers. Additionally, it enhances the growth of beneficial bacteria, such as *Akkermansia muciniphila*. Similarly, in the human cohort, higher propionate levels were inversely correlated with calcification scores, suggesting its protective role against vascular calcification [48].

In animal models, propionate reduced total and LDL cholesterol, decreased intestinal cholesterol absorption and lessened atherosclerotic lesions through immune-dependent regulatory T-cell and IL-10 increase. Accordingly, randomized, doble-blind placebo-controlled clinical trials showed that oral propionate supplementation significantly lowered LDL and total cholesterol levels, while increasing peripheral regulatory T-cells [49,50]. Although these findings might place propionate in a role of a novel therapeutic target for improving cholesterol homeostasis, short duration of clinical trials raises questions regarding the sustainability of propionate’s effects and potential negative feedback mechanisms over longer periods.

Studies in HF patients have revealed reduced fecal SCFA concentrations compared to healthy controls, particularly butyrate. This SCFA deficiency correlates with reduced abundance of SCFA-producing bacteria, including *Faecalibacterium prausnitzii*, and *Eubacterium rectale* [51,52]. The depletion of these beneficial bacteria and their metabolites may contribute to the pro-inflammatory state characteristic of HF [53].

Animal models have provided mechanistic insights into SCFA cardioprotection. Butyrate supplementation in rodent HF models reduced cardiac hypertrophy, fibrosis, and inflammation while improving cardiac function [54]. Propionate administration improved endothelial function and reduced blood pressure in hypertensive animal models [55]. These findings suggest that restoration of SCFA production through dietary fiber supplementation or targeted probiotic interventions may offer therapeutic benefits in HF.

### 4.3. Secondary Bile Acids

It is widely recognized that BAs are essential for cholesterol excretion. The deconjugation of primary BAs into secondary BAs—such as deoxycholic and lithocholic acids—occurs in the distal ileum through microbial enzymatic activity [56]. They function as signaling molecules through activation of nuclear receptors (farnesoid X receptor (FXR) and G-protein-coupled receptors (TGR5)), regulating glucose and lipid metabolism, inflammation, and energy homeostasis [57]. In vivo, the dual inactivation of Farnesoid X receptor (FXR) and Takeda G-protein-coupled receptor 5 (TGR5) exhibits aortic inflammation and atherosclerosis via nuclear factor κB (NF-κB) activation in LDL receptor knockout (LDLR KO) mice [58]. FXR activation preserves intestinal lining integrity and permeability, alongside with controlling inflammation and fibrosis. Further animal studies show that higher FXR expression may reduce myocardial ischemia–reperfusion injury, whereas FXR suppression is associated with increased myocyte apoptosis and infarct size [59]. Additionally, a link has been identified between FXR activation and the upregulation of angiotensin II type 2 receptor, which was shown to prevent salt-sensitive hypertension in rat models—suggesting potential therapeutic application [60].

Human studies on bile acid metabolites, such as deoxycholic acid, and ursodeoxycholic acid have investigated their associations with cardiometabolic disturbances, yielding contradictory results. A cohort analysis including 112 patients with moderate-to-severe chronic kidney disease revealed that elevated serum deoxycholic acid levels were positively correlated with higher baseline coronary artery calcification scores [61]. In contrast, in a randomized, double-blind, placebo-controlled crossover trial with 20 healthy male participants, ursodeoxycholic acid increased bile acid hydrophilicity but unexpectedly increased LDL cholesterol levels. Additionally, it may have antagonistic effects on FXR pathways, potentially inhibiting beneficial outcomes [62]. A multi-omics analysis conducted within the framework of the DIRECT-PLUS trial found positive associations between specific BAs, such as ursodeoxycholic acid, hyocholic acid, and lithocholic acid families and triglycerides or the total cholesterol/high-density lipoprotein cholesterol (TC/HDLc) ratio [63].

These findings highlight the complexity of bile acid interactions and the need for more comprehensive studies to explore effective interventions for managing LDL cholesterol levels. The limited participant numbers available for longitudinal analysis resulted in underpowered statistical tests and the use of non-fasting serum samples, and the demographic homogeneity of the study population limits the generalizability of the results. The cardiovascular effects of bile acids are complex and context-dependent. HF patients might exhibit altered bile acid profiles characterized by increased primary-to-secondary bile acid ratios. This impaired bacterial bile acid transformation may result from reduced abundance of bacteria capable of bile acid metabolism [64].

## 5. Microbiome–Drug Interaction in Heart Failure Pharmacotherapy

Intestinal bacteria play a crucial role in metabolizing both endogenous and exogenous substances, significantly influencing drug metabolism as orally administered drugs pass through the intestine. While the liver is the primary site for biotransformation, gut bacteria, particularly in the colon, can transform drugs into active, inactive, or toxic metabolites. The gut microbiota produces various enzymes that facilitate biotransformations like hydrolysis and reduction, directly impacting drug metabolism. They also affect pharmacokinetics by altering the bioavailability of medications and influencing the absorption of prodrugs from the upper intestine [65].

**Figure 3:**
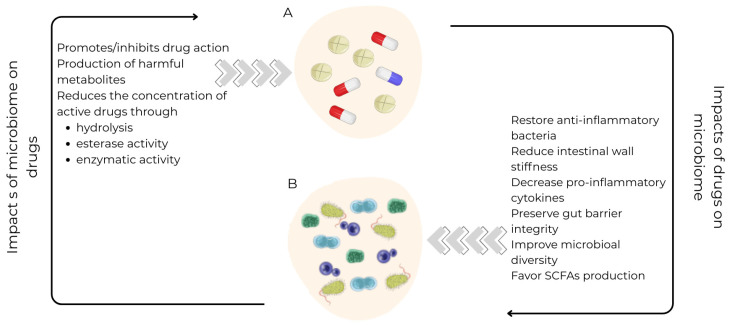
Bidirectional interactions between drugs and the gut microbiome. (**A**) Microbiome impacts on drugs: gut microbes can promote or inhibit drug action, produce harmful metabolites, and reduce the concentration of active drugs via hydrolysis, esterase activity, and other enzymatic transformations. (**B**) Drug impacts on the microbiome: drugs can restore anti-inflammatory bacteria, reduce intestinal wall stiffness, decrease pro-inflammatory cytokines, preserve gut barrier integrity, improve microbial diversity, and favor short-chain fatty acids (SCFAs) production. Bidirectional arrows indicate reciprocal influence between drug molecules and microbial communities. (Image provided by Servier Medical Art (https://smart.servier.com/) (accessed on 12 April 2026), licensed under CC BY 4.0 (https://creativecommons.org/licenses/by/4.0/) (accessed on 12 April 2026).

Advances in genome sequencing technologies and bioinformatics have enabled detailed description of the gut microbiome’s composition, genetic diversity, and its interaction with pharmaceutical agents [66]. Large-scale human cohort studies—including the Dutch LifeLines-DEEP cohort, the Belgium Flemish Gut Flora Project, and the TwinsUK cohort—have identified associations between various medications and changes in gut microbiome composition and function [67]. Many cardiovascular drugs rank among the top microbiome-associated pharmaceuticals, including statins, angiotensin-converting enzyme inhibitors, beta-blockers and antithrombotic agents [68] (Figure 3).

### 5.1. Renin-Angiotensin-Aldosteron System Inhibitors

Renin–angiotensin–aldosterone system blockade remains the cornerstone in heart failure management and is active throughout the gastrointestinal tract. Both angiotensin receptors—type I (AT1R) and type II (AT2R)—are expressed in epithelial villi, submucosal layers, muscular layers, the myenteric plexus and local vasculature [69]. Angiotensin receptor blockers (ARBs) and angiotensin-converting enzyme inhibitors (ACEIs) reduce intestinal wall rigidity, enhance villi length, decrease pro-inflammatory cytokine concentration, preserve gut barrier integrity, and improve microbial diversity—as pointed out in animal experiments [70]. In a recent study involving 55 hypertensive patients, Dong et al. assessed the effects of ACEI/ARB treatment on gut microbiome and metabolites, using a multi-omics approach combining 16S rRNA gene sequencing for gut microbiome analysis and fecal metabolomic analysis. Although the study used a cross-sectional design and had a small sample size, it suggested that RAAS inhibition reduces pathogenic bacteria such as *Enterobacter* and *Klebsiella*, while promoting beneficial ones like *Odoribacter*. Additionally, significant metabolomic shifts were observed, with higher levels of inositol in the well-controlled group [71]. It should be emphasized, however, that the cross-sectional design of the study limits the ability to establish causality or observe dynamic changes in microbial and metabolic features. It also lacks information on important cofounding factors, such as dietary habits, duration of medication and patient adherence.

In addition, the microbiome may play a significant role in the hydrolysis of quinapril and ramipril, reducing the concentration of the active drug, while others, like lisinopril, are less susceptible to microbial degradation [72]. These findings were revealed by Yang et al. in a research that combines both animal experimentation and human sample analysis. It integrates in vivo animal models, in vitro biochemical assays, and microbial sequencing techniques. The primary experimental design involved spontaneously hypertensive rats, demonstrating that the blood pressure lowering effect of quinapril was more notable in the antibiotic-treated group. This study pointed out that depletion of gut microbiota in vitro, specifically the reduction in *Coprococcus comes*, reduced the catabolism of esterified ACEIs, a phenomenon absent with non-ester ACEIs, like lisinopril. In addition, a human cohort consisting of 29 hypertensive patients demonstrated enrichment of the *Coprococcus comes* species. Still, a small sample size and lack of statistical significance limit the conclusive clinical relevance and generalizability of these human findings. Although animal models are valuable for mechanistic aspects, the findings may not directly apply to human physiology due to inherent species differences [73].

Finally, a few attempts have been made to investigate novel heart failure drugs, such as the sacubitril/valsartan combination and microbiome interactions, though these findings remain preliminary and may not be directly transferable in a clinical setting. Animal models have shown that sacubitril-/valsartan-treated diabetic mice exhibit reduced harmful bacterial genus like *Escherichia* and *Shigella*, and express increased proliferation of others with beneficial traits, such as *Lactobacillus*, *Bacteroides*, and *Parabacteroides*, potentially supporting gut barrier integrity and promoting SCFA production [74].

### 5.2. Beta-Blockers

Known as the fundamental treatment in heart failure, beta-blockers exert their effect through modulating the sympathetic nervous system, reducing morbidity and mortality in patients with heart failure [75]. Until recently, little was known about their interaction with the gut microbiome. In a 2024 population-based observational cohort study involving 134 participants diagnosed with cardiometabolic disease, Shearer et al. found that beta-blocker therapy contributed to the depletion of *Akkermansia muciniphila* and compromised the activity of *Egerthella lenta*, both of which are important for energy metabolism and immune regulation [76]. These results, however, need to be interpreted in the light of their correlational nature, which cannot definitely establish a causative relationship between beta-blocker use and gut microbiota changes. Additionally, there was a lack of detailed information regarding prescription duration, dosage and medication formulation.

### 5.3. Mineralocorticoid Receptor Antagonists (MRAs)

Furthermore, according to multiple animal-based studies, spironolactone also facilitates the restoration of SCFAs-producing bacterial species, supports the Firmicutes/Bacteroidetes ratio, and reduces systemic inflammation, thus helping maintain eubiosis [60,67]. The first evidence of spironolactone’s dysbiosis-reducing effect was provided by González-Correa et al. in an experimental research on spontaneously hypertensive rats (SHR) followed by a five-week treatment. As a result, administration of spironolactone lead to the restoration of acetate-producing *Bacteroides* and *Prevotella* genus on one hand and decreased the concentration of butyrate-producing bacteria on the other hand, especially from the *Eubacteriacea* family and *Clostridiales* order. In addition, they observed a reduction in sympathetic drive in the gut, a normalization of higher proportions of Th17 cells in mesenteric lymph nodes and Th17 infiltration in the aorta in SHR receiving spironolactone administration [70]. Thus, spironolactone has beneficial effects on gut dysbiosis and sympathetic tone; it is unclear whether these contribute to the overall antihypertensive effect.

### 5.4. Sodium-Glucose Cotransporter 2 Inhibitors (SGLT2i)

The search for novel therapeutic strategies addressing the complex pathophysiology of HF has led to the repurposing of SGLT2 inhibitors into cornerstone therapies for HF management. Their clinical efficacy has been demonstrated across the spectrum of left ventricular ejection fraction, consistently reducing HF hospitalizations and making improvements in cardiorenal outcomes regardless of the presence of diabetes. Furthermore, SGLT2 inhibitors produce microbiome changes across rodent models which commonly include enrichment of SFA-producing taxa and reduction in the *Firmicutes*/*Bacteroides* ratio [77]. However, human evidence of microbiome alterations is limited to small clinical cohorts; SGLT2i treatment has shown favorable shifts in SCFA producers and reductions in proinflammatory taxa. Unfortunately, robust trials linking microbiome shifts to clinical HF endpoints are not yet available. In a retrospective study of a comprehensive multi-omics approach, combining 16S rRNA gene sequencing and untargeted metabolomics including 135 individuals, Zhang et al. found significant alterations of gut microbial composition and circulating metabolic signature in HF patients treated with dapagliflozin. These changes involve an increase in beneficial bacteria like *Akkermansia*, *Collinsella*, and *Butyricicoccus* [78].

In addition, a randomized, open-label, two-arm clinical trial by Deng et al. found that patients with type 2 diabetes and several CVD risk factors treated with empagliflozin express higher microbiome diversity after one month of treatment. They reported a significant shift in favor of SCFAs-producing beneficial bacteria, including *Roseburia*, *Eubacterium*, and *Faecalibacterium* [79]. Although this study provides strong evidence for empagliflozin’s benefits, its open-label nature, small sample size, and short follow-up period are important considerations. Further research with larger, longer, and potentially blinded trials is needed to confirm and expand upon the long-term cardiovascular outcomes. Modulation of the gut microbiome appears to be one plausible mediator of SGLT2i cardio protection and a potential target for complementary therapies, but clinical translation remains preliminary.

### 5.5. Diuretics—In-Focus Loop Diuretics

There is little direct evidence that loop diuretics themself cause reproducible, specific shifts in gut microbial taxa. Most human and animal data describe microbiome changes associated with heart failure rather than with diuretic exposure per se. Therefore, reported compositional alterations in HF such as depletion of SCFA producers and increased *Proteobacteria*/*Actinobacteria* should not be assumed to be caused by furosemide without targeted studies [80]. The main microbiome-related safety signal for loop diuretics in the supplied literature is the pharmacokinetic-mediated increase in circulating TMAO, which has been associated with worse outcomes in HF; other microbiome-mediated adverse effects attributable to furosemide are not demonstrated. Observational HF studies associate higher TMAO with adverse prognosis, and loop diuretics increase circulating TMAO via reduced renal excretion, a mechanism shown in humans and validated in mice [81,82].

### 5.6. Anticoagulants

Oral anticoagulation therapy represents a critical part of HF treatment in those who associate atrial fibrillation or thrombotic diseases. Atrial fibrillation is the most common comorbidity in HF, affecting up to 40–50% of patients. Two major classes of oral anticoagulants are currently used in clinical practice. Vitamin K antagonists (VKAs) have been the standard of care for decades, acting by inhibiting the hepatic synthesis of vitamin K-dependent clotting factors. Despite their proven efficacy, VKAs are notoriously difficult to manage due to their narrow therapeutic window, multiple drug and food interactions and wide interindividual variability in anticoagulant response. Convergent preclinical and human evidence suggests microbiome contributions to VKA variability via vitamin K biosynthesis and community perturbation [83]. Although not directly related to HF, in a research including 200 patients who had undergone heart valve replacement and were treated with warfarin, stool samples taxonomic profiling via 16S rRNA sequencing revealed that high relative abundance of *Escherichia-Shigella* impairs responses to warfarin anticoagulation therapy. *Enterococcus*, on the other hand, enhances warfarin anticoagulation capacity. Furthermore, high responders showed significantly reduced gut microbial diversity compared to normal and low responders, as estimated by the Shannon index [84].

Direct oral anticoagulants (DOACs), including the factor Xa inhibitors apixaban, rivaroxaban, edoxaban, and the direct thrombin inhibitor dabigatran, have largely supplanted VKAs in many clinical settings owing to their more predictable pharmacokinetics, fixed dosing, and fewer dietary interactions. Human data regarding their interaction with the gut microbiome remain sparse, but observational studies in AF patients and preclinical animal models suggests that DOACs can also shift gut microbial composition by increasing opportunistic taxa such as *Streptococcus*, *Escherichia*, *Shigella*, and *Klebsiella*, on one hand, and partially preserving beneficial genera like *Bifidobacterium* and *Lactobacillus*, on the other hand [13,85]. In a rat model, rivaroxaban induced dose-dependent changes in microbial alpha and beta diversity that correlated with coagulation indices, suggesting that DOAC–microbiome interactions may have functional pharmacodynamic consequences. Nevertheless, antibiotic-mediated microbiome disruption has been shown to increase rivaroxaban bioavailability in animal models [70].

### 5.7. Antiplatelets

Aspirin is a widely used antiplatelet agent in the prevention and treatment of CVD, and acts by inhibiting cyclooxygenase-1 enzymes and prostaglandin synthesis. According to latest findings, aspirin causes gastrointestinal damage and toxicity in up to 90% of patients. Long-term aspirin treatment leads to alterations in gut microbiome composition, impacting bacterial genera like *Prevotella*, *Bacteroides*, *Barnesiella*, as well as the *Ruminococcaceae* family. In contrast, studies have suggested that dysbiosis may also influence aspirin metabolism [86,87]. For instance, *Parabacteroides goldsteinii* was identified by Li et al. to have a beneficial effect on restoring aspirin-related intestinal-layer damage by suppressing bile acid receptor FXR signaling. This study was a combined experiment using aspirin-treated mice and clinical research including healthy human subjects who received a 100 mg of aspirin treatment daily for 30 days. Stool samples were analyzed by whole-genome shotgun sequencing. They further established that the growth of *Parabacteroides goldsteinii* is inhibited by aspirin [88].

These findings suggest that probiotic or bile acid-based therapies could be valuable strategies for preventing aspirin-associated enteropathy. Nevertheless, the research primarily focused on *Parabacteroides goldsteinii* and its role, whilst other aspirin-suppressed species that may have potential functions in mitigating intestinal damage were not thoroughly explored. Clopidogrel, another platelet agent, has also been associated with a distinct microbial fingerprint and enhanced diversity, as shown in metagenomic analyses from large populational studies, including the Twins UK cohort [89].

### 5.8. Cardiac Glycosides

Digoxin, the most commonly used cardiac glycoside, is largely prescribed for the treatment of heart failure and atrial fibrillation. It has been widely known for its marked interindividual variability in terms of bioavailability [90]. Emerging evidence shows that digoxin can be metabolized by the gut microbiome into an inactive form, dihydrodigoxin, in around 10% of patients [90,91]. The bacteria responsible for this inactivation was identified in the early 1980s as *Eubacterium lentum*, a member of the *Actinobacteria* phyla, later renamed as *Eggerthella lenta* [91].

In a mechanistic and translational study, Haiser et al. used RNA sequencing to identify a two-gene cytochrome-encoding operon of *Eggerthella lenta*, referred to as the cardiac glycoside reductase, which showed significant upregulation in the presence of digoxin. These genes were claimed to potentially serve as a predictive microbial biomarker for digoxin inactivation. Moreover, they showed that the growth of *E. lenta* might be dependent on arginine levels, which both supports its proliferation and inhibits digoxin inactivation. Consequently, increasing arginine levels—whether through dietary intake or from a microbial source—may offer a reasonable strategy to prevent this undesired bacterial activity [92].

### 5.9. Statins

Statins have proven to be the most beneficial treatment for patients with hypercholesterolemia, both in primary and secondary prevention, significantly reducing cardiovascular morbidity and mortality [93]. Evidence from animal experiments and human studies show an interplay between statin therapy and the gut microbial landscape [94,95,96]. Sun et al., in a comparative analysis of gut microbiota including 202 hypercholesterolemic patients divided into a statin-sensitive (SS) and statin-resistant (SR) cohort, found that patients with extensive bacterial heterogeneity showed a better response to statin treatment. The SS group exhibited microbial genera known for cholesterol-lowering properties, namely *Lactobacillus*, *Bifidobacterium*, *Faecalibacterium* and *Eubacterium*, successfully lowering serum low-density lipoprotein (LDLc) levels below 100 mg/dL within three months [97]. However, they specifically focused on first-time statin users which might limit the generalizability of the results to those with prior or long-term statin exposure.

Likewise, Shi et al., in a two-sample Mendelian randomization study using the Genome-Wide Association study (GWAS) summary, including 18 340 European participants from 24 different cohorts, revealed genetic proxies for lipid-lowering drugs able to influence microbiome abundance. Therefore, overexpression of Niemann-Pick C1-Like 1 protein (NPC1L1) was associated with the increase in the *Egerthella* genus, 3-hydroxy-3-methylglutaryl-CoA reductase (HMGCR)-modulated LDL-cholesterol elevation with the *Pasteurellales* order and *Haemophilus* order. Similarly, a proprotein convertase subtilisin/kexin type 9 (PCSK9)-mediated increase in LDL-cholesterol also showed an association with a higher abundance of the *Terrisporobacter* genus [98]. Nevertheless, the analysis did not differentiate between the effects of individual drugs corresponding to each specific drug target. Due to reliance on summary sequencing data from large populations, the study lacks subgroup analyses of the effects of lipid-lowering drugs among patients with different chronic diseases.

Furthermore, a cross-sectional, observational study by Khan et al. suggested that atorvastatin may selectively restore anti-inflammatory bacteria, such as *Akkermansia muciniphilia* and *Faecalibacterium prausnitzii*, which in turn promote the growth of the *Oscillospira* genus. The study population included a s mall sample size divided into three groups, 15 untreated hypercholesterolemic patients, 27 atorvastatin-treated hypercholesterolemic patients, and 19 healthy subjects. By using 16S rDNA amplicon sequencing to evaluate the gut bacterial community, it reported that the atorvastatin-treated group demonstrated lower bacterial diversity compared to healthy participants, a significant reduction in pro-inflammatory taxa and enrichment of anti-inflammatory phyla [99]. While this study provides valuable insights into how atorvastatin treatment modulates the gut microbiota in hypercholesterolemic patients, its findings are limited by the lack of metabolomic data that would provide a more in-depth understanding of microbial influences on the specific actions of atorvastatin.

In contrast, in a randomized placebo-controlled double-blinded trial, Kummen et al. found only a modest effect of rosuvastatin use on gut microbiome composition on a short-term follow-up of two to four weeks. While the sample size consisting of 66 subjects limited the ability to detect subtle microbial changes, a notable increase in pro-atherogenic microbiome-derived metabolites was seen among patients with a poor HDL/LDL ratio improvement, suggesting a link between statin response and microbiome activity [100] (Table 1).

## 6. Discussion

Due to significant development in metagenomics and next-generation sequencing, there has been an escalation in gut microbiome research over the past two decades. Numerous preclinical and clinical studies have reported interrelation between changes in intestinal microbiome composition and its metabolic function. Although there is an undoubtful association between cardiovascular pathology and microbiome activity—highlighting the significance of the gut–heart axis—the complex, bidirectional dynamics and pathophysiological mechanisms involved in drug–microbiota interactions are far from straightforward [101,102]. Studies utilizing metagenomic sequencing techniques have uncovered correlations between medication consumption and gut microbial alterations. These interactions hold significant potential for enhancing personalized treatment strategies in HF management.

The evidence base for cardiovascular drug–microbiome interactions is predominantly composed of small cross-sectional human studies (n = 15–200) and animal models, with only two randomized controlled trials identified across nine major drug classes. Critical limitations include reverse causality in cross-sectional designs, inadequate control for confounding factors such as diet, comorbidities or polypharmacy, and limited mechanistic validation in humans. Unfortunately, the evidence appears to be low-quality for most drug–microbiome associations, with moderate quality emerging only for digoxin metabolism by *Eggerthella lenta*, SGLT2 inhibitor effects on SCFA-producing taxa, and loop diuretic impacts on TMAO levels.

ACEIs and ARBs not only improve gut health but also play essential roles in heart failure management by promoting beneficial bacteria like *Odoribacter* and reducing pathogenic strains. Research by Dong et al. underlines how RAAS inhibition preserves gut barrier function and microbial diversity. The mechanistic rationale is interesting, where angiotensin II promotes inflammation and barrier dysfunction, and RASS blockade may reverse these effects while enriching inflammatory taxa. However, the primary human evidence comes from cross-sectional studies with sample sizes ranging from 30 to 200 participants, where RAAS inhibitor users showed higher abundance of *Odoribacter* and *Prevotella* compared to non-users. These studies suffer from inadequate control for several confounders. It is well-known that RAAS inhibitor users typically have hypertension, diabetes, or heart failure—all of these conditions independently associated with gut dysbiosis. Likewise, diet, particularly sodium intake, was not systematically controlled, nor was polypharmacy.

The cross-sectional human studies cannot establish whether RAAS inhibitors cause microbiome changes or whether baseline microbiome differences influence drug response or disease progression. Animal models provide mechanistic support, demonstrating that ACE inhibitor treatment in hypertensive rats restores microbial diversity and reduces *Enterobacteriaceae* abundance. These models use genetically homogeneous animals in controlled environments, limiting generalizability to the heterogeneous human population with variable diet, comorbidities, and medication regimens.

The evidence for beta-blocker effects on the gut microbiome is among the weakest in cardiovascular pharmacology. The primary human study by Shearer et al. reported reduced *Akkermansia muciniphila* abundance in beta-blocker users compared to non-users in a cross-sectional analysis. *A. muciniphila* is a mucin-degrading bacteria associated with metabolic health, and its depletion could theoretically worsen metabolic dysfunction in HF patients. Nevertheless, this single small study provides insufficient evidence for clinical recommendations. Critical limitations include the tiny sample size (n = 28), which provides inadequate statistical power to detect true associations or control for confounders. Heart failure patients on beta-blockers typically have more severe disease, higher NYHA class, and greater polypharmacy burden, all factors independently associated with gut dysbiosis. The study did not control for diet, physical activity, or concurrent medications making it impossible to isolate beta-blocker effects. The cross-sectional design precludes causal inference: does beta-blocker use deplete *A. muciniphila*, or do patients with lower baseline *A. muciniphila* have more severe heart failure requiring beta-blocker therapy?

MRAs, such as spironolactone, seem to demonstrate a positive influence on gut microbiome diversity by restoring SCFA-producing bacteria. Research suggests that these medications facilitate a healthier microbiome, correlating with reduced systemic inflammation and enhanced cardiac outcomes in HF. Mechanistically, aldosterone promotes intestinal inflammation and fibrosis; accordingly, MRA blockade may reverse these effects while enriching SCFA-producing taxa such as *Faecalibacterium prausnitzii* and *Roseburia* species. While these findings seem mechanistically plausible and potentially clinically important, they require validation in large, prospective, adequately controlled trials.

The evidence for SGLT2 inhibitor effects on the gut microbiome is among the strongest in cardiovascular pharmacology, though still limited by methodological constraints. Two prospective studies by Zhang et al., n = 60 in heart failure patients, and Deng et al., n = 40 in type 2 diabetes patients, reported increased SCFA-producing bacteria, including *Faecalibacterium*, *Roseburia* and *Bifidobacterium* and elevated fecal SCFA concentrations after 3–6 months of empagliflozin or dapagliflozin treatment [78,79]. These studies provide stronger evidence than cross-sectional analyses by establishing temporal sequence: SGLT2 inhibitor initiation preceded microbiome changes. Mechanistically, SGLT2 inhibitors increase glucose delivery to the colon, providing substrate for bacterial fermentation and SCFA production. SCFAs exert anti-inflammatory effects, improve gut barrier function, and may contribute to the cardiovascular benefits of SGLT2 inhibitors. Despite this, critical limitations remain. Both studies were small (n = 40–60), single-center, and lacked placebo controls, limiting causal inference. Dietary intake was not rigorously controlled or monitored, despite the fact that diet is a dominant driver of SCFA-producing bacteria.

Furthermore, loop diuretics represent a unique case where the drug–microbiome interaction occurs primarily through altered renal excretion rather than direct microbiome modulation. Two independent studies—Latkovskis et al., n = 50, and Li et al., n = 30, demonstrated that loop diuretics decrease renal elimination of TMAO, leading to elevated plasma TMAO levels [80,81,82]. The main mechanism that stays at the base of these results was that loop diuretics inhibit the renal transporter responsible for TMAO excretion, leading to TMAO accumulation independent of microbiome composition changes. This represents a pharmacokinetic interaction rather than a direct drug–microbiome effect. Yet, both studies were small (n = 30–50) and observational, limiting causal inference. Loop diuretic users typically have greater fluid overload and worse renal function, all factors independently associated with elevated TMAO levels. The studies did not adequately control for dietary choline and carnitine intake, renal function, or concurrent medications. The cross-sectional design cannot establish whether loop diuretics cause TMAO elevation or whether patients with higher baseline TMAO have more severe disease requiring loop diuretic therapy. It remains unknown whether TMAO elevation is a causal mediator of HF progression or merely a biomarker of disease severity. Interventional studies manipulating TMAO are needed to establish causality.

The evidence for anticoagulant–microbiome interactions is limited and mechanistically unclear. Small cross-sectional studies (Wang et al., n = 50; Li et al., n = 60; Chen et al., n = 45) reported associations between warfarin or DOACs and gut microbiome composition. Wang et al. found that *Enterococcus* and *Escherichia-Shigella* abundance correlated with warfarin dose requirements, suggesting that these bacteria may influence warfarin metabolism or vitamin K availability. Li et al. reported that DOAC users had altered microbiome composition compared to non-users, with reduced *Bacteroides* and increased *Firmicutes* [85,86,87]. Nevertheless, the proposed underlying mechanistic is unclear. Warfarin inhibits vitamin K-dependent clotting factors, and gut bacteria produce vitamin K2 (menaquinones), potentially influencing warfarin dose requirements. However, the contribution of bacterial vitamin K2 to systemic vitamin K status is uncertain, and dietary vitamin K1 (phylloquinone) is the dominant source. For DOACs, which do not interact with vitamin K, the mechanistic basis for microbiome effects is even less clear.

On the other hand, antiplatelet agents, particularly aspirin, can induce dysbiosis, reducing the number of beneficial bacteria such as *Prevotella* and *Bacteroides*, as seen in studies focusing on long-term aspirin use. The evidence for aspirin–microbiome interactions is limited but mechanistically noteworthy. Li et al. (n = 80 mice, n = 30 humans) demonstrated that aspirin induces gut dysbiosis and intestinal damage, but *Parabacteroides goldsteinii* administration protected against aspirin-induced gastrointestinal toxicity through bile acid metabolism. This represents one of the few studies with mechanistic validation across animal models and human samples.

Another notable drug–microbiome interaction involves cardiac glycosides. The digoxin–*Eggerthella lenta* interaction represents the strongest and most mechanistically validated drug–microbiome interaction in cardiovascular pharmacology. The evidence spans four decades, beginning with Lindenbaum et al.’s 1981 observation that antibiotic therapy restored digoxin bioavailability in patients with reduced drug levels [91]. This is particularly relevant in HF therapy, where maintaining consistent digoxin concentrations is crucial for effective symptom management. Haiser et al. provided mechanistic insight, identifying the *cgr* operon in *E. lenta*, responsible for digoxin reduction to inactive metabolites [92]. However, limitations remain. The prevalence of digoxin-inactivating *E. lenta* strains varies across populations and is incompletely characterized. The studies were small (n = 20–50) and did not assess whether routine microbiome screening to identify *E. lenta* carriers improves clinical outcomes. The interaction is most relevant in the minority of patients with high *E. lenta* abundance and may be less important in the era of declining digoxin use.

Conversely, some CV medication like statins, in particular atorvastatin, increase beneficial bacteria, including *Lactobacillus* and *Bifidobacterium*, as noted in studies by Sun et al. and Khan et al. Patients with higher microbial diversity tend to exhibit better lipid-lowering responses, emphasizing the importance of microbiome composition in predicting drug efficacy. In HF, where managing lipid levels is critical for cardiovascular risk reduction, this relationship suggests that analyzing gut microbiota composition could guide statin therapy more effectively. The evidence for statin–microbiome interactions is moderately strong, with consistent findings across multiple studies. Sun et al. (n = 100 hyperlipidemic patients) reported that statin responders had higher baseline abundance of *Lactobacillus* and *Bifidobacterium* compared to non-responders. Khan et al. (n = 50 hypercholesterolemic patients) demonstrated that atorvastatin treatment increased *Lactobacillus* and *Bifidobacterium* abundance after 3 months. Kummen et al. (n = 10 healthy volunteers) found that rosuvastatin altered gut microbiome composition in a small randomized crossover trial [99,100,101,102]. Diet, particularly fiber and fat intake, profoundly influences both lipid levels and microbiome composition, yet was not rigorously controlled in most studies. Statin users may differ systematically from non-users in dietary habits, physical activity, and comorbidities, all factors affecting the microbiome. The studies did not assess whether microbiome-guided statin selection improves clinical outcomes compared to standard therapy.

## 7. Limitations

The exact mechanistic interplay between pharmacogenomic and microbiome-mediated interactions remains insufficiently elucidated, mainly due to challenges arising in microbiome research [103]. As mentioned previously, many studies on microbial metabolism of several CV drugs often involve small sample sizes and rely on animal models, limiting the generalizability of these findings to human populations. Many findings are drawn from small, specific cohorts, which may not represent broader populations, potentially limiting the applicability of the conclusions. Unfortunately, the evidence base for cardiovascular drug–microbiome interactions is characterized by critically underpowered studies. Across nine major drug classes, the median sample size for human studies is n = 50 (range: 10–200), with the majority (>70%) having fewer than 100 participants. These small sample sizes provide inadequate statistical power to detect true associations, control for confounders, or identify subgroup effects.

Most of the studies’ cross-sectional design limits the ability to conclude causality and may overlook confounding factors such as diet and medication adherence that can influence microbiome composition. The correlational nature of findings does not establish causation, highlighting the need for more robust, controlled trials to confirm specific relationships. These results are essentially associative, rather than casual, limiting the strength of clinical conclusions. The predominance of cross-sectional designs is above 80% of human studies, representing a fundamental limitation for causal attribution. Cross-sectional studies measure drug exposure and microbiome composition at a single time point, precluding determination of temporal sequence: does the drug cause microbiome changes, or do baseline microbiome differences influence drug prescription, response, or disease progression? Reverse causality is a critical concern. Patients with certain microbiome profiles may have more severe disease, leading to prescription of specific medications. For example, heart failure patients with gut dysbiosis may have worse functional status, prompting more aggressive pharmacotherapy. Observing altered microbiome composition in these patients does not establish that the medications caused the changes—the dysbiosis may have preceded and contributed to disease severity.

Clinical translation of current findings is further impeded by the lack of precise methodological design, small sample size, short follow-up and disregard of various confounding factors, such as dietary habits [104,105]. Diet is the dominant driver of gut microbiome composition, yet the majority of studies did not systematically assess or control for dietary intake. This represents a critical methodological flaw that undermines causal inference. Specific dietary components profoundly influence the microbiome taxa implicated in drug–microbiome interactions. Fiber intake is the primary determinant of SCFA-producing bacteria—the same taxa reportedly increased by SGLT2 inhibitors and MRAs. Choline and carnitine intake determines TMAO production—the metabolite reportedly elevated by loop diuretics. Vitamin K intake influences warfarin dose requirements and may confound associations between anticoagulants and vitamin K-producing bacteria.

Furthermore, HF patients have high rates of comorbidities that independently affect the gut microbiome: diabetes, chronic kidney disease, obesity, hypertension, and atrial fibrillation. Each of these conditions are associated with distinct microbiome alterations, and failure to adequately control for comorbidity burden. Clinical translation of current findings is further impeded by disregard of various confounding factors such as polypharmacy, drug–drug interactions, medication dosage and formulation, as well as individual variability in drug response. Extended-release formulations may have different effects on the microbiome compared to immediate-release formulations due to altered drug delivery to different intestinal segments. Dose–response relationships are rarely assessed, limiting mechanistic insight.

Another important feature is represented by the limitations and generalizability of animal studies. Although animal models provide valuable mechanistic insights and enable controlled experiments impossible in humans, rodent microbiomes differ fundamentally from human microbiomes in composition, diversity, and functional capacity. Specific bacterial species implicated in human drug–microbiome interactions, such as *Eggerthella lenta* for digoxin, are often absent or rare in rodent models. Laboratory animals are genetically homogeneous, housed in controlled environments, and fed standardized diets—conditions that do not reflect the genetic diversity, environmental exposures, and dietary heterogeneity of human populations. Drug doses used in animal models often exceed human equivalent doses, and pharmacokinetics differ substantially between species. The translational gap from animal models to humans is evident in the literature. Many drug–microbiome interactions observed in rodent models have not been validated in human studies, and some human associations lack animal model support. While animal models are essential for mechanistic hypothesis generation, clinical translation requires validation in adequately powered human studies (Supplementary Materials, Table S1 and Table 2).

## 8. Conclusions and Future Directions

Overall, cardiovascular disease management is shifting towards a more personalized care, potentially integrating genetic, epigenetic, and microbiome-based approaches. Pharmacomicrobiomics represents a promising frontier in the new era of individualized medicine. The current evidence base reveals that while drug–microbiome interactions in cardiovascular medicine are mechanistically plausible and potentially clinically important, the quality of evidence remains predominantly low for most drug classes. Only three interactions have moderate quality evidence, such as digoxin metabolism by *Eggerthella lenta*, SGLT2 inhibitor effects on SCFA-producing bacteria, and loop diuretic effects on TMAO levels. These represent proof-of-concept examples demonstrating that pharmacomicrobiomics is feasible and potentially clinically relevant.

Therapeutic modulation of the gut microbiome represents a promising strategy to enhance cardiovascular drug efficacy and minimize adverse effects. Potential interventions include the administration of probiotics in order to supplement *Parabacteroides goldsteinii* to protect against aspirin-induced gastrointestinal toxicity, *Lactobacillus* and *Bifidobacterium* to enhance statin efficacy, *Akkermansia muciniphila* to restore metabolic dysregulation in beta-blocker users, as well as multi-strain probiotic formulations to restore SCFA-producing bacteria. Likewise, several prebiotics may find their usefulness within this setting. For instance, dietary fiber supplementation to increase SCFA production and support beneficial bacteria, dietary interventions, like the mediterranean diet to promote beneficial bacteria and SCFA production, choline and carnitine restriction to reduce TMAO production in loop diuretic users, vitamin K consistency in warfarin users. Even fecal microbiota transplantation (FMT) from healthy donors to restore microbiome diversity in severe dysbiosis might be a future strategy. Postbiotic interventions might be interesting as well, such as direct SCFA supplementation to bypass microbiome dysfunction.

In conclusion, due to the extensive inter-individual variability of the microbiome makeup, influenced by lifestyle, diet, environment, medical conditions and polypharmacy, major challenges arise when studying the microbiome. Future work should focus on well-designed studies with systematic patient selection and standardized sample collection to reduce the overload of these variables and facilitate the translational potential of findings into clinical application. Importantly, it must be emphasized that, at present, no microbiome-based biomarker or therapeutic intervention has been validated for clinical use in heart failure management, and none should be recommended outside of a research context. The future outlook is the identification of specific bacterial genes and phyla in order to anticipate drug metabolism, or the development of new biomarkers aimed to lead treatment decisions. Exploring the unique microbial profile of an individual may expand medical treatments with strategies able to modify the patient’s microbiome. Eventually, specific probiotics, prebiotics, or even postbiotics might become beneficial to enhance drug efficacy and safety in HF treatment.

## Figures and Tables

**Table 1:** Microbial changes induced by heart failure medication.

Drug Effect	Microbial Strain	Level of Evidence	Rationale
ACEI/ARB	↓ *Enterobacter* ↓ *Klebsiella* ↑ *Odoribacter*	LOW	Cross-sectional studies (n = 30–200); confounders not controlled
Sacubitril/Valsartan	↓ *Escherichia* ↓ *Shigella* ↑ *Lactobacillus* ↑ *Bacteroides* ↑ *Parabacteroides*	LOW	Limited cross-sectional/observational evidence only
Beta-blockers	↑ *Streptococcus* ↑ *Lactobacillus* ↓ *Akkermansia muciniphila* ↓ *Eggerthella lenta*	LOW	Single small study (n = 28); inadequate confounder control
Spironolactone	↑ *Bacteroides* ↑ *Prevotella* ↓ *Eubacteriacea* ↓ *Clostridiales*	LOW	Mechanistically plausible but no large prospective trials
Empagliflozin	↑ *Roseburia* ↑ *Eubacterium* ↑ *Faecalibacterium*	MODERATE	Prospective study (n = 60); temporal sequence established
Dapagliflozin	↑ *Akkermansia* ↑ *Collinsella* ↑ *Butyricicoccus*	MODERATE	Cross-sectional (n = 135) with 16S rRNA sequencing; consistent findings
DOACs	↑ *Streptococcus* ↑ *Escherichia* ↑ *Shigella* ↑ *Klebsiella* ↓ *Ruminococcus*	LOW	Small cross-sectional studies (n = 45–60); mechanism unclear
Aspirin	↓ *Parabacteroides goldsteinii* ↓ *Prevotella* ↓ *Ruminococcaceae* ↓ *Bacteroides* ↓ *Barnesiella*	LOW	Limited human data (n = 30); mostly animal model evidence
Statins	↑ *Lactobacillus* ↑ *Bifidobacterium* ↑ *Faecalibacterium* ↑ *Eubacterium*	MODERATE	Multiple study designs (RCT, cohort, Mendelian randomization); consistent findings

↑ Increase in microbial abundance ↓ Decrease in microbial abundance.

**Table 2:** Relevant articles of clinical studies.

Study	Year of Publication	Study Design	Results	Evidence Gaps and Future Insights
Dong et al. [71]	2022	A cross-sectional analysis including patients with hypertension to evaluate the effects of ACEI/ARBs on gut microbiome and metabolites.	ACEI/ARBs therapy reduces pathogenic bacteria like *Enterobacter* and *Klebsiella* while increasing beneficial ones such as *Odoribacter*.	Research is needed to monitor dynamic changes in microbial and metabolic features between well-controlled hypertensive patients and healthy subjects using ACEI/ARBs.
Zhang et al. [78]		Cross-sectional designed research on a total of 135 individuals with HF, comprising 84 patients treated with dapagliflozin and 51 receiving conventional therapy. Gut microbial communities were characterized using 16S rRNA gene sequencing.	The dapagliflozin group showed increased *Prevotella*, *Akkermansia*, *Collinsella*, and *Fusobacterium*, as well as decreased *Bacteriodes*, *Parabacteriodes* and *Bifidobacterium*.	The limited sample size and single-center design may affect the generalizability of results across diverse HF populations Cross-sectional design prevents establishing causal relationships between dapagliflozin, gut microbiota, and metabolomic profiles. Longitudinal follow-up and animal experiments are needed to confirm causality. The long-term and mechanistic dimensions of dapagliflozin’s gut–metabolic effects remains unknown.
Deng et al. [79]	2022	Randomized, open-label, two-arm clinical trial which included treatment-naive patients with type 2 diabetes and cardiovascular risk factors.	Significant reductions were found in glycated hemoglobin levels in both empagliflozin and metformin groups. Empagliflozin increased beneficial SCFA-producing bacteria and reduced harmful strains.	Larger sample sizes and longer follow-up periods are needed to investigate the underlying mechanisms of empagliflozin-related microbiome changes. Different SGLT2 inhibitors might also have impact on gut microbiome composition.
Shearer et al. [76]	2024	A population-based cohort study, involving 134 middle-aged adults diagnosed with cardiometabolic disease, focusing on the relationship between medication use and gut microbiota composition.	46 associations were identified between microbial composition and single medications, including β-blockers and statins depleting *Akkermansia muciniphila*. Increasing medication use correlated negatively with α-diversity in gut microbiota among participants.	Future research could utilize fecal metabolomics profiling to confirm functional changes in gut microbiota. Further exploration of the relationship between medications and gut microbiota at genus and species levels is warranted.
Lin Wang et al. [84]		Clinical investigation focused on the influence of gut microbiota on the response to warfarin treatment in 200 heart valve replacement patients, classified into low responder, high responder and normal responder groups.	Gut microbiota diversity and relative abundance differed significantly among warfarin responder groups. *Escherichia-Shigella* was enriched in low responders, impairing warfarin response. *Enterococcus* was enriched in high responders, enhancing warfarin anticoagulation capacity.	Patients had first undergone heart valve replacement and taken warfarin for only 8 weeks. Relatively small sample size. Fecal samples were collected after antibiotic and anticoagulant use, which might devalue clinical application.
Wan Li et al. [13]		Original research article that recruited 89 patients hospitalized for atrial fibrillation, divided in patients receiving long-term OAC treatment and patients not receiving OAC. A total of 40 volunteers were also included as control group.	OACs reduced pro-inflammatory *Ruminococcus* but increased potential pathogenic taxa, such as *Streptococcus*, *Escherichia-Shigella*, and *Klebsiella*.	16S rRNA gene sequencing provides taxonomic composition but lacks functional analysis. The exact signaling mechanisms through which gut microbiota interacts with OACs was not determined. Confounding factors like other medications, diet, and lifestyle may introduce bias. Additional large cohort studies and functional research are needed.
Sun et al. [97]	2018	Clinical trial involving hypercholesterolemic patients treated with atorvastatin. It compares the composition of gut microbiota between statin-sensitive and resistant groups.	Gut microbiota composition differs significantly between statin-sensitive and statin-resistant patients. The statin-sensitive group exhibited higher biodiversity compared to resistant group. Increased *Lactobacillus*, *Eubacterium, Faecalibacterium*, and *Bifidobacterium*. Decreased proportion of *Clostridium*.	The impact of genetic polymorphisms on statin pharmacodynamics remains a significant area of research. Further studies could assess how different bacterial taxa influence statin efficacy and dosage adjustments.
Shi et al. [98]	2024	A Mendelian randomization two-sample design, which utilizes genetic variants to estimate the impact of lipid-lowering medication on gut microbiota diversity.	Different genetic proxies for lipid-lowering drugs affects the abundance of gut microbiota. Some have been associated with an increase in the genus *Eggerthella*. Others were linked to the order *Pasteurellales* and the genus *Haemophilus*.	Studies using individual-level data are needed for comprehensive insights on associations. Investigating the effects of lipid-lowering drugs across different patient subgroups is necessary.
Khan et al. [99]	2018	A cross-sectional observational study that compares gut microbiota analyses among three groups: untreated hypercholesterolemic patients, atorvastatin-treated patients, and healthy subjects.	Atorvastatin treatment increased anti-inflammatory bacteria, *A. muciniphila* and *F. prausnitzii*, and decreased the levels of proinflammatory taxa, such as members of *Proteobacteria* phylum, in hypercholesterolemic patients.	Future studies should focus on the specific mechanisms by which gut microbiota influences lipid metabolism and inflammation.
Kummen et al. [100]	2020	A randomized controlled trial assessing the effects of rosuvastatin on gut microbiome composition.	Rosuvastatin treatment showed no significant changes in gut microbial diversity. Reduced potential to metabolize TMAO precursors.	Re-analyzing TMAO-related metabolites in previous statin trials could provide valuable insights. Exploring the mechanisms behind the pleiotropic effects of statins may enhance understanding of their impact on the microbiome.

## Data Availability

Not applicable.

## Supplementary Materials

The following supporting information can be downloaded at: https://www.mdpi.com/article/10.3390/life16050823/s1, Table S1: Relavant articles of preclinical findings.

## Abbreviations

The following abbreviations are used in this manuscript:

CVD	Cardiovascular disease
HF	Heart failure
MetaHIT	Metagenomics of the Human Intestinal Tract
TNF-α	Tumor necrosis factor alfa
IFN-γ	Interferon gamma
TMAO	Trimethylamine-N-oxide
SCFAs	Short-chain fatty acids
SBAs	Secondary bile acids
MACE	Major Adverse Cardiac Events
BNP	B-type natriuretic peptide
FXR	Farnesoid X receptor
TGR5	Takeda G protein-coupled receptor 5
LDL	Low-Density Lipoprotein
HDL	High-Density Lipoprotein
RAAS	Renin–angiotensin–aldosterone system
AT1R	Angiotensin 1 receptor
AT2R	Angiotensin 2 receptor
ARBs	Angiotensin receptor blockers
ACEIs	Angiotensin receptor inhibitors
