# Supplementary figures and images for: Microbiome-Associated Drug Response Variability in Heart Failure Treatment
Source: Life (Basel). 2026 May 15;16(5):823. doi: 10.3390/life16050823 (PMC13208838; doi:10.3390/life16050823)

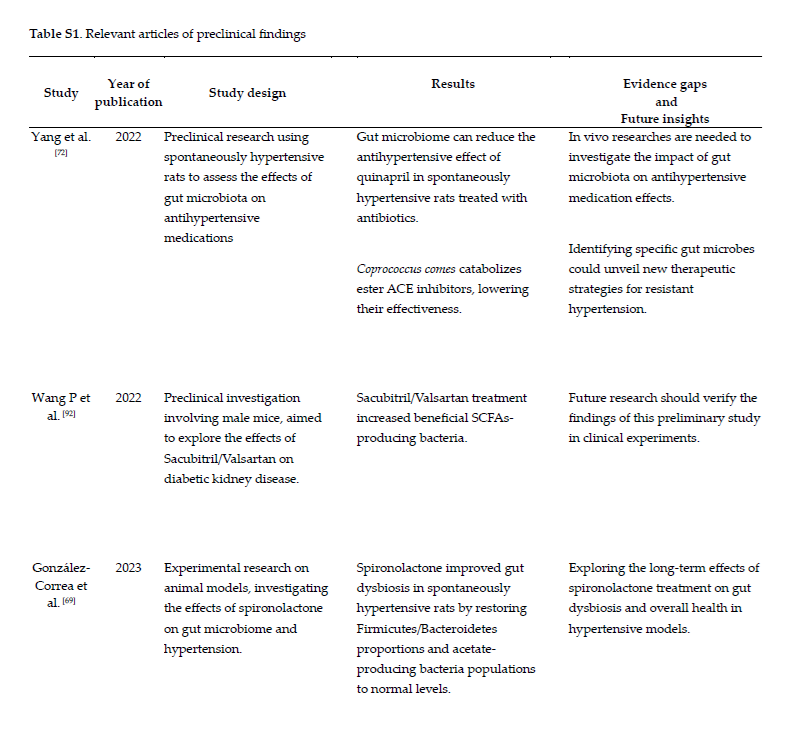

Supplement: Supplementary file 1 [file life-16-00823-s001.zip › Table S1, page1.png]

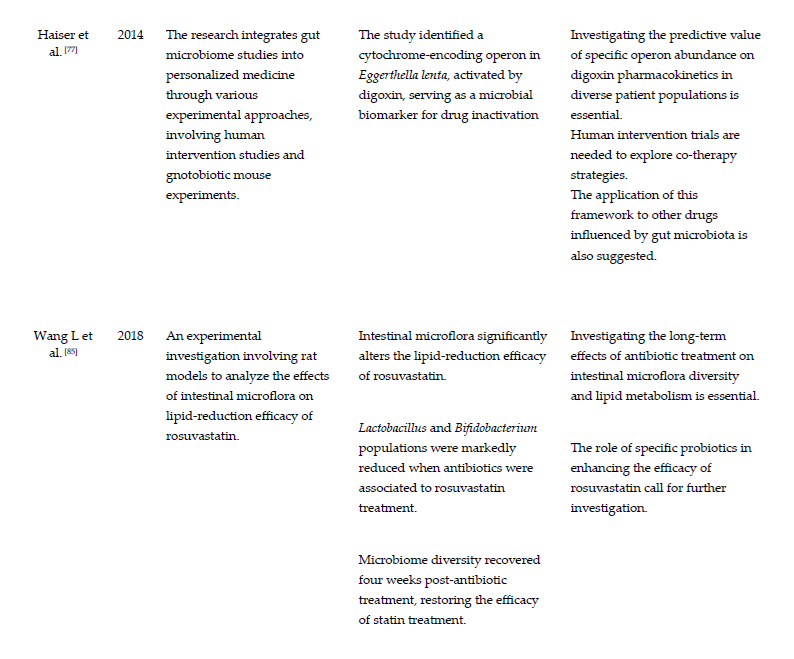

Supplement: Supplementary file 1 [file life-16-00823-s001.zip › Table S1, page2.png]
